# Storage Properties and Shelf-Life Prediction of Fresh-Cut Radishes Treated by Photodynamic Technology

**DOI:** 10.3390/foods13152367

**Published:** 2024-07-26

**Authors:** Sijia Ruan, Tong Zhu, Changzhou Zuo, Jing Peng, Liwang Liu, Weijie Lan, Leiqing Pan, Kang Tu

**Affiliations:** 1 College of Food Science and Technology, Nanjing Agricultural University, Nanjing 210095, China; 2 College of Food Science and Technology, Yunnan Agricultural University, Kunming 650201, China; 3 College of Horticulture, Nanjing Agricultural University, Nanjing 210095, China

**Keywords:** photodynamic treatment, minimally processing, storage quality, shelf life, kinetic model

## Abstract

Fresh-cut radishes are susceptible to quality loss and microbial contamination during storage, resulting in a short shelf life. This study investigated the effects of photodynamic technology (PDT) on fresh-cut radishes stored at 4 °C for 10 d and developed appropriate models to predict the shelf life. Results showed that curcumin-mediated PDT maintained sensory acceptability, color, and firmness, decreased weight loss, and increased ascorbic acid and total phenolics of samples by inactivating polyphenol oxidase and peroxidase, resulting in improved antioxidant capacity and quality. The total bacteria count in samples was significantly (*p* < 0.05) reduced by 2.01 log CFU g^−1^ after PDT and their shelf life was extended by 6 d compared to the control. To accurately predict the shelf life, the kinetic models based on microbial growth were established, while weight loss, b* value, firmness, and ascorbic acid were selected as representative attributes for developing quality-based prediction models through correlation analysis. Modeling results showed prediction models based on ascorbic acid best fitted PDT-treated samples, while the modified Gompertz model based on bacteria growth was the best for control and samples treated by sodium hypochlorite. This study suggests that PDT is promising in extending the shelf life of fresh-cut radishes, and using critical indexes to establish the prediction model can provide a more reliable shelf-life estimation.

## 1. Introduction

Consumer demands for fresh-cut products have grown rapidly as a result of changes in lifestyle and health awareness in recent years [1]. Fresh-cut radishes are popular products due to their added values of health-promoting properties, freshness and convenience [2]. However, oxidative stress caused by cutting process might result in their quality deterioration during storage, including color changes, water loss, texture softening, and microbial contamination [1]. Consequently, various preservation methods have been applied to prolong the shelf life of fresh-cut radishes, including edible coatings, sodium hypochlorite (NaClO) solution, and modified atmosphere packaging [3,4,5,6]. However, the aforementioned methods have their own limitations. Among them, edible coating can reduce soluble solids content of radish shreds and modified atmospheres has little effect on ascorbic acid of fresh-cut turnips [2,7], while NaClO treatment may introduce safety concerns though it can effectively inhibit browning and microbial growth. Therefore, there is great interest in exploring a safe, low-cost, and efficient postharvest technology with less impact on the storage properties for fresh-cut radish products.

As a non-thermal and environmentally friendly method, photodynamic technology (PDT) has been efficiently applied in preserving fruits and vegetables. Current studies have shown that PDT can inhibit the growth of foodborne pathogens on the surface of foods including lettuce, fresh-cut pears and pineapples [8,9,10]. The antimicrobial effects of PDT are mainly due to photosensitizer that is excited by light with specific wavelengths to generate reactive oxygen species (ROS), such as singlet oxygen, hydrogen peroxide, or highly active superoxide radicals, which cause microbial death [1]. Curcumin is a natural food additive and is widely used as a photosensitizer in foods due to its non-toxic photodegradation products [11]. Compared with photo treatment alone, curcumin-mediated PDT treatment can inhibit enzymatic browning and microbial growth due to inactivation of relevant enzyme and bacteria [12]. Currently, the effect of PDT on storage quality and shelf life of fresh-cut radish products is unclear.

Accurate shelf-life prediction is crucial for estimating food quality and ensuring food safety for fresh-cut products [13]. Microbial growth is commonly used as the basic index to establish models such as the modified Gompertz model, the Huang model, and the Baranyi model to estimate the shelf life of foods [13,14,15]. Mathematical models based on physicochemical indicators can also be used to predict food quality loss during storage [16]. For example, zero- and first-order kinetics are commonly used to monitor the quality deterioration of various foods (such as vegetables, fruits, and aquatic products) under different storage conditions [16,17,18]. However, most of the aforementioned research predicted the shelf life of products based on models either by bacteria or quality indicator only. Very limited research has compared the food shelf life obtained by predictive models based on both microbial aspects and quality aspects. When assessing the shelf life of fresh-cut products, both microbial growth and quality changes need to be fully considered. To the best of our knowledge, there is no information available for the shelf-life prediction for fresh-cut radishes.

Thus, the aims of this study are as follows: (i) explore the effects of curcumin-mediated PDT on the quality of fresh-cut radishes stored at 4 ± 1 °C; (ii) determine the critical attributes that affect the shelf life of fresh-cut radishes through correlation analysis with microorganisms; and (iii) develop and compare predictive models based on quality and safety aspects. This study provides a practical approach for the preservation and shelf-life prediction of fresh-cut radishes.

## 2. Materials and Methods

### 2.1. Preparation of Fresh-Cut Radishes

Fresh white radishes (*Raphanus sativus* L.) were harvested and transported immediately from the National Modern Agriculture Industry Park (Taixing, China) to the laboratory in 2 h, and those with similar size and maturity and free of mechanical injury were selected. After washing, samples were peeled and sliced into a thickness of 4 mm using a slicer (Lejie Electric Co., Guangzhou, China). For those treated by PDT, the sample slices were immersed in 30 µmol L^−1^ curcumin solution (Shanghai Acemc Biochemical Technology Co., Ltd., Shanghai, China) [10] for 15 min in dark and then exposed to a blue light-emitting diode (LED) (Guixiang Co., Ltd., Weifang, China) for 24.5 min with the intensity of 100 μmol·m^−2^·s^−1^.These PDT processing parameters were optimized by artificial neural network model and genetic algorithms according to our previous study [19]. The radish slices under NaClO treatment were immersed in 200 μL L^−1^ NaClO solution for 2 min according to Chen et al. [20] and sample without any treatment was used as control (CK). After being air-dried, samples (150 g) were randomly packed in a polyethylene box (Nanjing Shoude Co., Ltd., Nanjing, China) with plastic wrap (Wuxi Xindi Paper Plastic Products Co., Ltd., Wuxi, China) covered. Nine boxes of samples were evaluated for each treatment and the first evaluation was performed 4 h after treatment. All samples were stored at 4 ± 1 °C and 90% relative humidity for ten days.

### 2.2. Sensory Evaluation

The sensory quality of fresh-cut radishes (color, aroma, off-odor, texture, and overall acceptance) was evaluated by a panel (*n* = 10) of assessors who received a 2-h session training per week for 4 successive weeks to ensure they were well trained for the sensory evaluation of fresh-cut radishes. The assessors were selected and trained according to ISO 8586: 2023. Samples (*n* = 9) labeled with three-digited codes were served randomly. Sensory evaluation was conducted following the criteria as described by Derossi et al. [21] with slight modification: Color was scored as follows: 1 = severe surface browning, 3 = slight surface browning and 5 = no browning, white and bright surface. Intensity of aroma was described as follows: 1 = no aroma, 3 = faint aroma, 5 = fresh aroma. Intensity of off-odors was graded as follows: 1 = severe off-odors, 3 = moderate off-odors, 5 = no off-odors. Texture was scored as follows: 1 = extremely soft, 3 = slight soft, 5 = extremely crispy. Overall acceptance was described as follows: 1 = unacceptable, 3 = fairly acceptable, 5 = extremely acceptable. The total average scores were calculated, and the higher scores, the better sensory quality of the products.

### 2.3. Determination of Weight Loss, Firmness, and Color

The weight of fresh-cut radishes was recorded at each storage time, and weight loss rate was calculated referring to the method of Helland et al. [2]. The firmness of samples was determined using the TMS-Pro texture analyzer (FTC, Washington, DC, USA) equipped with a flat probe (diameter: 3.5 cm). The probe was pressed down at 1 mm s^−1^, with a deformation degree of 50%, and the starting force was set to 0.6 Newton (N). The maximum force was recorded as sample firmness (N) and determined by 9 replicates independently. For color measurement, three positions of each radish slice were randomly selected and values of L*, a*, and b* were recorded by a colorimeter (CR-400, Konica Minolta, Tokyo, Japan). The images of samples were taken at day 0, day 8 and day 10 by a camera (Cannon, Tokyo, Japan) under the same camera mode with the flash off and the same focal length and aperture.

### 2.4. Determination of Ascorbic Acid and Total Phenolics

Ascorbic acid content was determined using the method slightly modified from Chen et al. [22]. Samples (2 g) were ground in 5 mL of 0.05 M oxalic acid ethylene diamine tetraacetic acid (EDTA) solution and then homogenized. The supernatant was collected by centrifugation at 8000× *g* for 15 min. The reaction solution included 1 mL of the supernatant, 4 mL of oxalic acid EDTA solution, 0.5 mL of 3% partial phosphoric acid-acetic acid solution, 1 mL of 5% H_2_SO_4_ solution, and 2 mL of 5 (NH_4_)_2_MoO_4_ solution. The mixture was made to a volume of 25 mL, incubated in the 80 °C water bath for 1 h, cooled to room temperature, and measured at 760 nm. The results were expressed as in milligrams per kilogram on a fresh weight basis.

The total phenolics was determined following the method by Huang et al. [23]. Two grams of samples were weighed and extracted in 30 mL of 60% (*w*/*w*) ethanol for 2 h in the dark. The mixture was centrifuged at 10,000× *g* for 20 min. The reaction solution consisted of 1 mL of supernatant, 0.5 mL of the Folin-Phenol reagent, 2 mL of Na_2_CO_3_ (1.0 M), and 6.5 mL of distilled water. After shaking, the mixture was rested at 25 °C for 1 h and determined at 760 nm. The TPC was presented as gallic acid equivalents in milligrams per kilogram on a fresh sample weight basis.

### 2.5. Determination of Antioxidant Activities

The 1,1-diphenyl-2-picrylhydrazyl (DPPH) radical scavenging capacity was measured as described by Cheng et al. [24] with modification. Combining 2 mL of supernatant gathered from the TPC extraction with 2 mL of DPPH solution (0.1 mM) for a 30-min reaction and the mixture was measured at 517 nm. DPPH radical scavenging capacity was calculated as follows:

(1) $$\mathrm{DPPH}\;\mathrm{radical}\;\mathrm{scavenging}\;\mathrm{capacity}=\left(1-\frac{A_{1}}{A_{0}}\right)\times 100\%$$

where *A*_1_ was 2 mL of gathered supernatant mixed with 2 mL of DPPH solution (0.1 mM); *A*_0_ was 2 mL of DPPH solution mixed with 2 mL of 60% (*w*/*w*) ethanol.

The enzyme extract of polyphenol oxidase (PPO) and peroxidase (POD) was prepared based on the methods of Wang et al. [25] with modification. Samples (2 g) were ground in 5 mL of extraction buffer (0.1 M, pH 5.5 acetate buffer solution, 1 mM polyethylene glycol, 1% Triton X-100, and 4% polyvinyl pyrrolidone) and then centrifugated at 10,000× *g* for 30 min. The supernatant (0.1 mL) was collected, and reacted with 4.0 mL acetate–sodium acetate buffer solution (0.1 M, pH 5.5) and 1.0 mL catechol (0.1 M). The absorbance was measured every 10 s at 420 nm within 4 min and the change value per minute was used as one unit (U) of PPO activity. POD activity was performed using 0.1 mL of enzyme supernatant, 3 mL of guaiacol solution (0.025 M), and 0.5 mL hydrogen peroxide (0.5 M). The absorbance was measured every 10 s at 470 nm within 4 min and the change value per minute was regarded as one unit of POD activity.

### 2.6. Microbiological Analysis

Fresh-cut radishes (25 g) were homogenized with 225 mL of sterile saline for 1 min. The homogenate was serially diluted (1:10, sterile saline solution), then 1 mL of sample was pour plated to plant count agar and incubated at 37 °C for 48 h. The plate count method was used to count the colonies and the results are expressed as logarithm of colony forming units per gram (log CFU g^−1^) of fresh weight.

### 2.7. Developing Kinetic Modeling for Microbial Growth and Quality Changes of Fresh-Cut Radishes during Storage

Kinetics models used for the growth of microorganisms in fresh-cut radishes are the modified Gompertz, modified Logistic, Baranyi, and Huang models [26,27,28,29]. The models equations are presented in order of (2)–(7).

The modified Gompertz equation:

(2) $$N_{t}=N_{0}+\left(N_{\max}-N_{0}\right)\times \exp\left\{-\exp\left[\frac{\mu_{\max}e}{N_{\max}\;-\;N_{0}}\left(\lambda -t\right)+1\right]\right\}$$

The modified Logistic equation:

(3) $$\begin{array}{c} N_{t}=\frac{N_{\max}}{1+(\frac{N_{\max}}{N_{0}}-1)\exp\left(\mu_{\max}\left(\lambda -t\right)\right)} \end{array}$$

The Baranyi equation:

(4) $$\begin{array}{c} N_{t}=N_{0}+\mu_{\max}A(t)-In\left\{1+\frac{\exp\left[\mu_{\max}A(t)\right]-1}{\exp\left(N_{\max}-N_{0}\right)}\right\} \end{array}$$

(5) $$A(t)=t+\frac{1}{\mu_{\max}}\mathrm{In}\left[\exp\left(-\mu_{\max}t\right)+\exp\left(-h_{0}\right)-\;\exp(-\mu_{\max}t-h_{0})\right]$$

The Huang equation:

(6) $$\begin{array}{c} N_{t}=N_{0}+N_{\max}-In\left[\exp\left(N_{0}\right)+(\exp\left(N_{\max}\right)-\exp\left(N_{0}\right))\times \exp(-\mu_{\max}B_{t})\right] \end{array}$$

(7) $$\begin{array}{c} B_{t}=t+\frac{1}{4}\mathrm{In}\left[\frac{1+\exp(-4\left(1-\lambda \right))}{1+\exp\left(4\lambda \right)}\right] \end{array}$$

where *N_t_*, *N*_0_, and *N*_max_ are the logarithm values of the count of microorganisms at time *t*, initial count, and maximum count (log CFU g^−1^), respectively; *µ*_max_ is the maximum specific growth rate (d^−1^); *λ* is the lag time for microbial growth (d); *t* is the storage time (d); and *h*_0_ can be calculated as *λ* × *µ*_max_.

The kinetic reaction models used for describing the change of quality attributes of radish samples were the zero-order and first-order reaction model [30]. The equations are shown as follows:

(8) $$\begin{array}{c} zero-order:A=A_{0}-kt \end{array}$$

(9) $$\begin{array}{c} first-order:A=A_{0}e^{-kt} \end{array}$$

where *A* is the quality indicator corresponding to storage time (t); *A*_0_ is the initial quality indicator; *t* is the storage time (d); and *k* is the reaction rate (d^−1^).The coefficients of determination (*R*^2^), the residual sum of squares (*RSS*) and root mean square errors (*RMSE*) were used to determine the performance of the kinetic models.

### 2.8. Statistical Analysis

Data from all experiments were analyzed using SPSS software (version 26, IBM SPSS Inc., Chicago, IL, USA). At least three repetitions were performed for each experimental analysis and all data were represented as the means ± standard deviation. Analysis of variance (ANOVA) with Duncan test was performed to determine the significant difference at level of *p* = 0.05. Origin software (version Pro 2022b, OriginLab, Northampton, MA, USA) was used for graphing.

## 3. Results and Discussion

### 3.1. Effects of PDT on the Quality of Fresh-Cut Radishes during Storage

#### 3.1.1. Sensory Analysis

**Figure 1:**
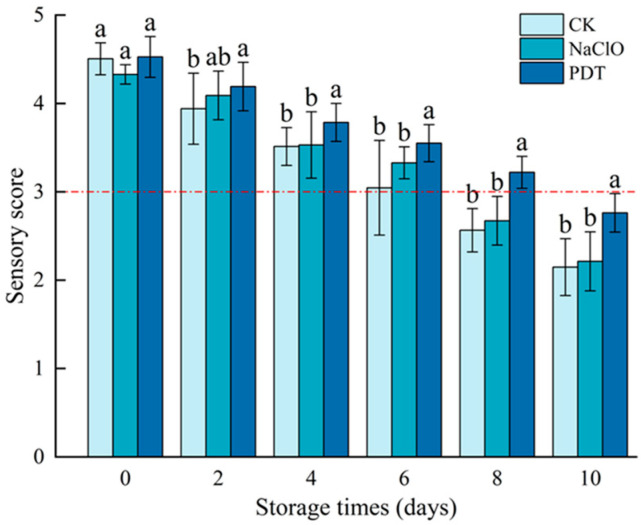
Sensory scores of fresh-cut radishes after PDT treatment. The red dotted line represents sensory acceptability limit. CK—control, NaClO—NaClO treatment, PDT—PDT treatment. Different letters indicate significant differences between different treatments at the same storage time (*p* < 0.05).

The results of sensory evaluation for control, NaClO-, and PDT-treated fresh-cut radishes are as shown in Figure 1. Sensory scores of all samples decreased as storage time was prolonged. However, samples treated by PDT had a significantly higher score than the control and those treated by NaClO (*p* < 0.05), which may be attributed to the positive effect of PDT on preserving the color and texture of fresh-cut radishes, and this was confirmed by our subsequent experiments [8]. Similar results were found in the study of Zou et al. [10], who observed higher appearance scores of fresh-cut pineapples treated with PDT during 5-day storage at 4 °C. According to Di et al. [31], who used the same sensory evaluation criteria as in this study, the fresh-cut radishes will lose commercial value if the sensory score is less than 3. Based on this, the shelf life of control, NaClO-, and PDT-treated samples was 6, 6, and 8 d, respectively, indicating that the sensory quality of PDT-treated samples was better than those treated by NaClO.

#### 3.1.2. Color, Weight Loss, and Firmness

**Figure 2:**
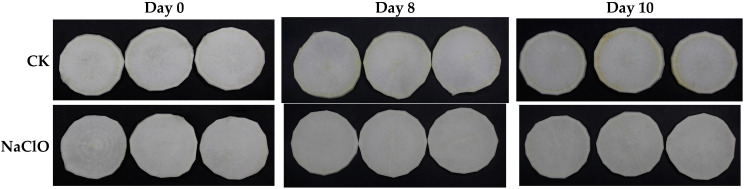

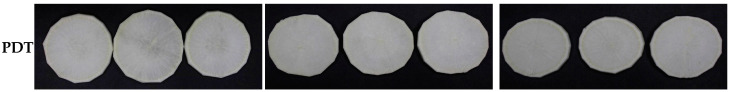
Effect of PDT treatment on the appearance of fresh-cut radishes on day 0, day 8, and day 10. CK—control, NaClO—NaClO treatment, PDT—PDT treatment.

Color can directly reflect the freshness of foods, which in turn greatly affects consumer decisions [32]. As shown in Table 1, a gradual decrease in L* value and an overall increase in b* value was observed, indicating surface browning in all samples during storage. It can also be seen from Figure 2 that the edges of the control turned yellow apparently at the end of storage. PDT-treated samples had the highest L* value during storage and preserved better visual appearance than the control. All treated samples exhibited significantly lower b* values than the control (*p* < 0.05). Samples treated by PDT exhibited higher b* values than those treated by NaClO, which was due to the residual curcumin on the product surface and can be degraded in a couple of hours during storage. After 2-day storage, no significant differences of b* values were observed between these two treated samples. Similar results were observed in fresh-cut pineapple where higher curcumin concentration caused the higher b* value than the control at the initial storage [10]. The a* values of all samples were relatively stable during storage, ranging from 0.63 to 0.93, which was due to the absence of pigments such as anthocyanins, carotenoids, or chlorophyll in the white radishes [5]. L* value and b* value can reflect the degree of browning [33]. During storage, PDT-treated samples had a higher L* value, a lower b* value, and better appearance, suggesting its effectiveness in inhibiting browning, which was verified by our later experiment.

At the end of 10-day storage, weight loss of PDT-, NaClO-, and non-treated samples was 1.5%, 2.6%, and 4.1%, respectively, and PDT-treated samples showed the lowest values during the whole storage. Respiration of all samples was accelerated by cutting, leading to an increase of water loss and even weight loss [34]. PDT could delay weight loss in fresh-cut radishes, which is likely due to the inhibitory effect of PDT on the enzymatic activities involved in the respiration process, therefore resulting in the reduction of water loss from respiration [35].

**Figure 3:**
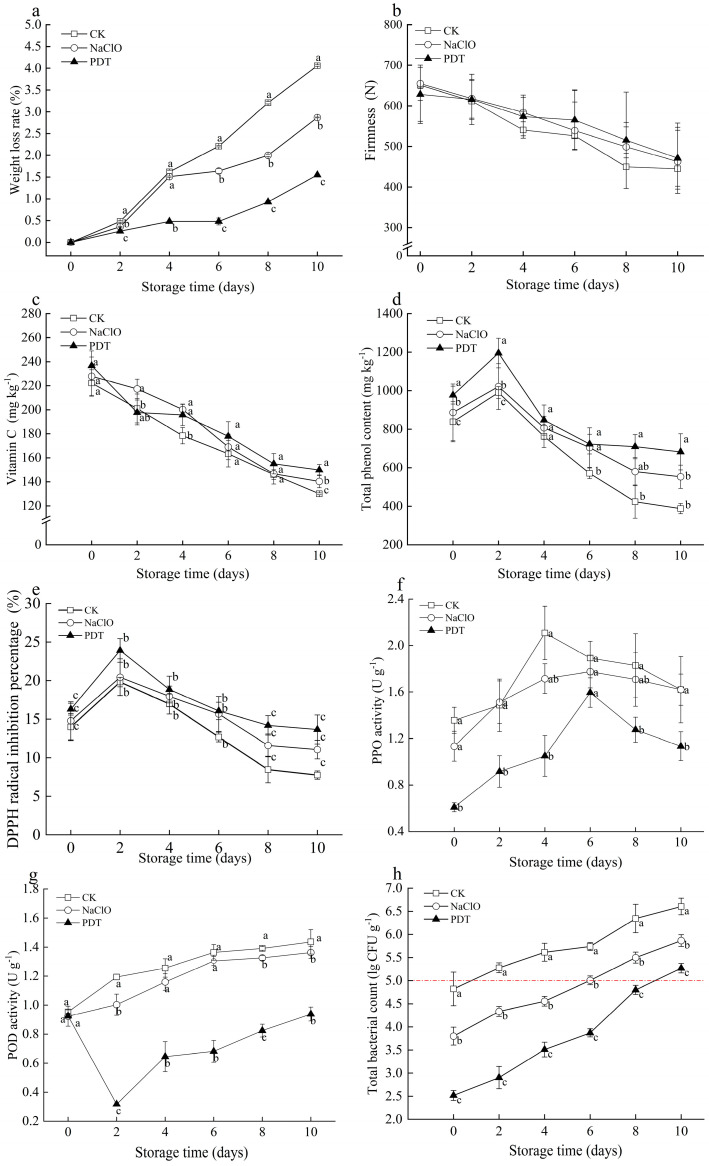
Effect of PDT treatment on the changes of weight loss (**a**), firmness (**b**), ascorbic acid (**c**), total phenol content (**d**), DPPH radical scavenging capacity (**e**), PPO activity(**f**), POD activity (**g**), and the total bacterial count (**h**) of fresh-cut radishes. The red dotted line represents the limit for total bacteria count. CK—control, NaClO—NaClO treatment, PDT—PDT treatment. Different letters indicate significant differences between different treatments at the same storage time (*p* < 0.05).

Firmness is a vital marketable attribute for evaluating the texture of fresh-cut radishes [36]. The firmness of all tested samples showed a consistent and notable decline during storage (Figure 3b). The maximum firmness of PDT-treated samples decreased by 30.62%, less than the decrease in untreated samples (34.50%) and NaClO-treated samples (31.86%) at the end of storage. Moisture loss and microorganisms growth can be inhibited by PDT, thereby delaying the decrease in firmness [37]. However, these positive impacts of PDT on maintaining the firmness of fresh-cut radishes were limited due to the possible undesirable cell structural changes caused by photochemical effects [38].

#### 3.1.3. Ascorbic Acid, Total Phenolic Content, and DPPH Radical Scavenging Capacity

Ascorbic acid and phenolic compounds are main bioactive compounds in fresh-cut products, which are vital for their antioxidant capacity to absorb and neutralize free radicals [20]. Ascorbic acid of fresh-cut radishes gradually decreased during storage (Figure 3c). Although no significant difference was observed between the untreated and treated samples, samples treated by PDT showed less ascorbic acid loss compared to those treated by NaClO. At the end of storage, ascorbic acid in PDT-treated samples was 149.9 mg kg^−1^, while that in non- and NaClO-treated samples was 130.0 mg kg^−1^ and 140.3 mg kg^−1^, respectively. Zou et al. [10] also observed higher ascorbic acid in fresh-cut pineapples treated by PDT, compared with control. The decrease in ascorbic acid was related to enzymes that can oxidize ascorbic acid. Ascorbic acid is oxidized by ascorbate oxidase to dehydroascorbic acid in the removal of excessive hydrogen peroxide [39], and peroxide can convert dehydroascorbic acid into ascorbic acid and hydrogen peroxide. The dual effects of curcumin (polyphenolic antioxidants) and blue light illumination during PDT treatment can inhibit the activities of the aforementioned enzymes and produce primary and secondary metabolites that accumulated ascorbic acid [40].

As shown in Figure 3d, total phenol content (TPC) in all samples increased initially and decreased subsequently during storage. PDT-treated samples showed the highest content of TPC during the storage. The same result was observed by Yu et al. that fresh-cut potatoes treated with PDT had higher TPC [41]. The initial increase of TPC in all samples was attributed to oxidative stress by cutting [42], while the increase of TPC in PDT-treated samples was related to blue light exposure during PDT treatment. Light exposure such as white-blue LED and UV-C can promote the biosynthesis of phenolics during postharvest period [43]. This explained the less degradation of total phenolics in PDT-treated samples caused by biodegradation and catalytic oxidation [41]. Therefore, the PDT-treated samples were less susceptible to browning, which was consistent with the obtained color results (Table 1).

Changes in DPPH radical inhibition of all samples were similar to the trend of total phenol content (Figure 3e). Compared to control and NaClO-treated samples, samples treated with PDT had higher DPPH radical scavenging capacity. The initial increasing trend can be explained by resistance to abiotic stress stimulated by cutting. The antioxidant capacity of fresh-cut radishes can neutralize and scavenge free radicals and other reactive oxygen species, resulting in a subsequent decrease in DPPH radical scavenging capacity and total phenolic content. This implies that PDT can improve the antioxidant capacity of fresh-cut radishes during storage.

#### 3.1.4. PPO Activity and POD Activity

PPO and POD are essential in enzymatic browning, resulting in color changes of produce [44]. As shown in Figure 3f, the PPO activity of all treatments gradually increased and then decreased. Compared to control and NaClO-treated samples, the PPO activity of samples after PDT treatment immediately significantly decreased by 55.2% and 46.3%, respectively. The change of PPO activity was closely attributed to the biodegradation and catalytic oxidation of total phenolics in the presence of oxygen [45]. PDT-treated samples exhibited the lowest PPO activity, most likely due to the destruction of PPO enzyme structure by LED illumination [40].

An overall increasing trend was observed in the POD activity of fresh-cut radishes during storage (Figure 3g). The POD activity of PDT-treated samples decreased at early storage and increased thereafter. PDT treatment significantly inhibited the activities of POD (*p* < 0.05) due to the effects of PDT on the breakdown of secondary spatial structure of POD enzymes [41]. The oxidation of phenolics in samples by PPO and POD was accelerated with storage time, leading to increased browning [42]. The PDT-treated samples exhibited lower PPO and POD activities and higher retention of total phenols. These results were confirmed with aforementioned color measurement results and antioxidant capacity improvements, such that PDT can effectively maintain the lightness, ascorbic acid, and total phenolics, therefore preventing samples from browning by reducing the oxidation and browning of phenolic compounds.

### 3.2. Effects of PDT on the Microbial Growth of Fresh-Cut Radishes during Storage

The total bacteria count (TBC) was significantly reduced in fresh-cut radishes after PDT- or NaClO-treatments immediately, but gradually increased during storage (Figure 3h). In particular, the TBC of PDT-treated samples was significantly lower than the other samples (*p* < 0.05). According to the National Standard of China [46], the maximum allowable microbial amount for fresh-cut products is 10^5^ CFU g^−1^. Based on this criteria, PDT-treated samples exceeded the microbial limit on the 10th day of storage, much longer than the untreated samples (2 d) and NaClO-treated samples (6 d). The inactivation effect of PDT is dependent upon the parameters of processing and food characteristic. Lin et al. have reported that the TBC of Hami lemons treated by curcumin (50 µmol L^−1^) with 60 min irradiation exceeded 10^5^ CFU g^−1^ at 7 d [37]. The effect of PDT on reducing bacteria is related to abundant ROS, which is mainly targeted at cellular DNA [47]. ROS produced by curcumin through blue light illumination attack a variety of biomolecules like proteins, causing protein degradation and even cell membrane damage [8]. These results show that PDT treatment had good antimicrobial effects on fresh-cut radishes and extended their shelf life.

### 3.3. Correlation between Quality Indexes and Microbial Count in Fresh-Cut Radishes during Storage

**Figure 4:**
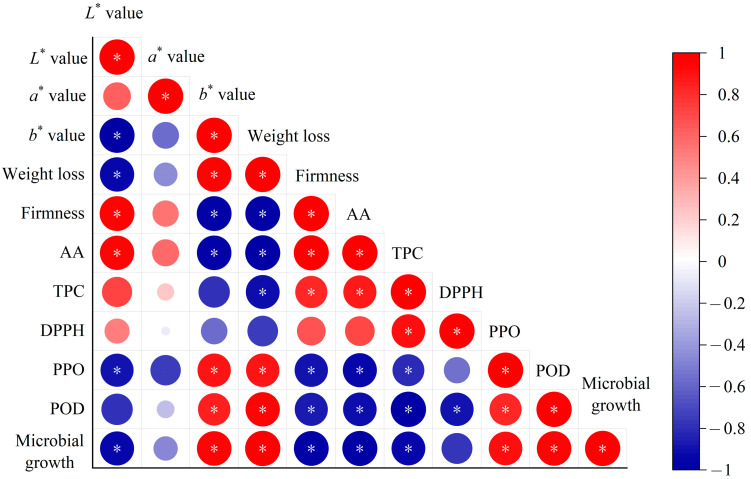
Correlation analysis between quality attributes and the TBC in fresh-cut radishes during storage. Notes: AA, ascorbic acid. TPC, total phenol content; DPPH, DPPH radical scavenging capacity; *: *p* < 0.05.

Modeling the changes of external and internal qualities such as color, firmness, and ascorbic acid content can be used to evaluate the deterioration process of products [16]. At present, there is no specific standard for determining acceptable limits for the quality attributes of fresh-cut vegetables, because consumer acceptance of each quality attribute is dependent upon food characteristics, compositions, and other factors [48]. During storage, quality attributes of fresh-cut vegetables are closely related to the activities of microorganisms. Generally, product quality deteriorates more slowly than the spread of microbial contamination [49], and the deterioration rate of each quality attribute varies during storage. Since it is common to monitor the state of microorganisms to ensure the safety of perishable foods, it is important to select key quality indicators that are most related to microorganisms to develop kinetic models for shelf-life prediction. The correlation between quality indexes and microbial count in fresh-cut radishes was evaluated using Pearson’s correlation analysis matrix (Figure 4). It can be seen that firmness, L* value, ascorbic acid, and TPC show significantly negative correlation with the TBC. In contrast, b* value, weight loss, and PPO enzyme are significantly positive correlated with the TBC. This indicates that with the growth of microorganisms, quality attributes of firmness, ascorbic acid, and TPC decreased, while b* values, weight loss, and PPO activity increased. A similar correlation was found in strawberries [50]. These highly correlated indicators with TBC can help to develop more effective quality prediction models. According to the Pearson correlation coefficients, the most related quality attributes (weight loss, firmness, b* value, ascorbic acid, and TPC) with *R* values > 0.9 were selected as key quality indexes for the shelf-life prediction of fresh-cut radishes.

### 3.4. Kinetic Models and Shelf-Life Determination for Fresh-Cut Radishes

#### 3.4.1. Kinetic Models for Microbial Growth

The performance of the different mathematical models used to describe the microbial growth in fresh-cut radishes are summarized in Table 2. Generally, the modified Gompertz model fitted better with the TBC in NaClO- and PDT-treated samples, with the highest *R*^2^ values of 0.987 and 0.982, the lowest *RSS* values of 0.017 and 0.052, and *RMSE* values of 0.091 and 0.162, respectively. Recent studies have shown that the modified Gompertz equation can be applied to simulate the growth curves of microorganisms in cherry tomatoes [51] and fresh-cut potatoes [52]. For the controls, the prediction accuracy of all developed models were similar, with *R*^2^ ranging from 0.989 to 0.992. PDT and NaClO treatments may exhibit distinct effects on microbial growth, potentially influencing the performance of predictive models. Overall, the modified Gompertz model can provide accurate estimations for the shelf life of all fresh-cut radishes based on microbial growth.

#### 3.4.2. Kinetic Models for Quality Attributes

The zero- and first-order kinetic models were used to monitor the deterioration in quality of fresh-cut radishes (Table 3). The results show that the performance of zero- and first-order reaction model varied depending on quality attributes. The zero-order reaction fitted better with weight loss, firmness, and b* value, while the first-order reaction described ascorbic more accurately. However, both zero-order and first-order kinetic models had poor fit on the changes of TPC in fresh-cut radishes. Similar results have been reported in some studies, confirming that the zero-order reaction model is suitable for monitoring the quality changes of cherry tomatoes and pineapples [16,51]. These findings show the effectiveness of the zero-order kinetic model in evaluating the variations in certain quality attributes of fresh-cut radishes.

#### 3.4.3. Shelf-Life Prediction

Microbial growth and quality properties can affect the shelf life of fresh-cut products [18]. Thus, defining the acceptable limit of quality and safety is important for controlling food safety and meeting consumer expectations [53]. According to aforementioned results, the shelf life of fresh-cut radishes under different treatments was determined within the limitation of the total bacteria count (*N_t_* < 5 log CFU g^−1^) and the sensory evaluation (sensory score < 3). Based on this, the results suggested that the shelf life for the control, NaClO-, and PDT-treated samples was less than 2 d (around 1.5 d), 6 d, and 8 d, respectively. As shown in Table 2, the predicted values of fresh-cut radishes obtained from the modified Gompertz model was 1.5 d for the control, 6.0 d for those treated by NaClO, and 9.1 d for those treated by PDT, which were in accordance with the experimental results obtained from microbial growth.

To replace sensory evaluation and further simplify shelf-life prediction, quality attributes that represented overall consumer acceptability were selected to establish kinetic models. Cut-off criteria for each of these attributes mentioned above were determined as the corresponding average values of 6 d for the control, 6 d for samples treated by NaClO, and 8 d for those treated by PDT according to sensory evaluation. The predicted shelf life of fresh-cut radishes based on quality attributes was obtained using the best-fit reaction order models and was summarized in Table 3. The results show that the predicted shelf life for fresh-cut radishes varied among the models using different quality attributes. The predicted shelf life based on ascorbic acid were closer to sensory shelf life of all fresh-cut radishes, indicating that the first-order kinetic model built by ascorbic acid content had a better accuracy in shelf-life prediction. However, the prediction models based on microbial growth offered better reliability than those based on quality attributes for the control and NaClO-treated samples, implying a quick microbial contamination than the quality degradation considering both safety and quality aspects when coming to shelf-life prediction. This was attributed to the fact that the shelf life of PDT-treated samples determined by the limit on the total bacteria count was longer than that determined by sensory evaluation. In fact, different quality attributes of fresh-cut radishes may decline at different rates during storage, resulting in a relatively large discrepancy for the predicted shelf-life values. Thus, it is important to determine representative quality attributes for the overall acceptance of the products by consumers.

## 4. Conclusions

This study investigated the effects of PDT on quality and microorganisms in fresh-cut radishes during storage and established shelf-life prediction models of fresh-cut radishes based on their quality attributes and microbial growth. The results show that PDT effectively improved antioxidant activity in fresh-cut radishes through inhibiting PPO and POD activities, as well as maintained higher ascorbic acid and phenolic contents, which may enhance the stability of cell interstitial structure. The sensory quality of fresh-cut radishes treated by PDT was also well maintained, and PDT significantly extended their shelf life by 6 d compared to the untreated samples. Different quality attributes (weight loss, firmness, b* value, and ascorbic acid) were used to establish the shelf-life prediction models for fresh-cut radishes after different treatments. Among them, the prediction model based on ascorbic acid was more accurate for samples treated with PDT, with an acceptable limit of 162.50 mg kg^−1^, which can take the place of sensory analysis for fresh-cut radishes. In contrast, safety-based models can provide a more conservative and precise shelf-life prediction for the control and NaClO-treated samples. This study suggests that taking both microbial and quality indicators into consideration is crucial to develop shelf-life prediction models for fresh-cut radishes by different treatments. Further experiments can be conducted to develop a comprehensive prediction model including all key safety- and quality-related parameters for shelf-life prediction of fresh-cut products.

## Figures and Tables

**Table 1:** Effects of PDT on CIE L*, a*, and b* values of fresh-cut radishes during storage.

Treatments		Storage Time (Days)					
		0	2	4	6	8	10
L* values	CK	69.63 ± 0.97 ^a^	65.30 ± 1.97 ^b^	65.08 ± 2.13 ^ab^	64.75 ± 1.28 ^b^	64.43 ± 1.63 ^b^	62.52 ± 1.81 ^ab^
	NaClO	66.22 ± 0.84 ^b^	64.70 ± 0.99 ^b^	64.58 ± 0.98 ^b^	64.53 ± 1.53 ^b^	63.50 ± 0.82 ^b^	62.43 ± 1.90 ^b^
	PDT	69.04 ± 0.79 ^a^	67.68 ± 1.20 ^a^	66.18 ± 0.63 ^a^	65.97 ± 2.27 ^a^	64.78 ± 0.45 ^a^	63.23 ± 2.47 ^a^
a* values	CK	−0.63 ± 0.05 ^a^	−0.87 ± 0.06 ^a^	−0.85 ± 0.11 ^a^	−0.70 ± 0.16 ^b^	−0.83 ± 0.13 ^a^	−0.80 ± 0.17 ^a^
	NaClO	−0.68 ± 0.09 ^a^	−0.80 ± 0.12 ^a^	−0.80 ± 0.13 ^ab^	−0.90 ± 0.06 ^a^	−0.88 ± 0.11 ^a^	−0.77 ± 0.08 ^a^
	PDT	−0.77 ± 0.20 ^a^	−0.90 ± 0.04 ^a^	−0.75 ± 0.07 ^b^	−0.89 ± 0.08 ^a^	−0.93 ± 0.05 ^a^	0.80 ± 0.07 ^a^
b* values	CK	0.97 ± 0.15 ^c^	3.32 ± 0.13 ^a^	3.22 ± 0.17 ^a^	3.65 ± 0.53 ^a^	3.75 ± 0.33 ^a^	4.40 ± 0.46 ^a^
	NaClO	1.68 ± 0.21 ^b^	2.23 ± 0.24 ^b^	2.63 ± 0.79 ^b^	3.03 ± 0.46 ^b^	3.33 ± 0.39 ^b^	3.40 ± 0.74 ^b^
	PDT	3.17 ± 0.22 ^a^	2.38 ± 0.14 ^b^	2.85 ± 0.55 ^b^	3.10 ± 0.29 ^b^	3.30 ± 0.18 ^b^	3.53 ± 0.35 ^b^

Notes: CK—control, NaClO—NaClO treatment, PDT—PDT treatment. Different superscript letters in the same column indicate significant differences (*p* < 0.05).

**Table 2:** Estimated model parameters for microbial growth in fresh-cut radishes under different treatments and the predicted safety-based shelf life.

Types of Models	Treat-ments	*N*_0_ (log CFU g^−1^)	*N*_max_ (log CFU g^−1^)	*µ*_max_ (d^−1^)	*λ* (d)	*R* ^2^	*RSS*	*RMSE*	Predicted Shelf Life (d)
Modified Gompertz model	CK	4.691	6.912	0.431	0.087	0.989	0.010	0.070	1.5
	NaClO	3.790	5.701	0.752	0.240	0.987	0.017	0.091	6.0
	PDT	2.258	7.732	0.758	0.623	0.982	0.052	0.162	9.1
Modified Logistic model	CK	4.820	6.739	0.439	0.642	0.991	0.011	0.075	1.6
	NaClO	3.470	5.791	0.725	1.080	0.928	0.110	0.235	6.2
	PDT	2.530	7.267	0.702	0.993	0.980	0.058	0.170	9.0
Baranyi model	CK	4.832	6.558	0.544	1.715	0.992	0.008	0.063	1.5
	NaClO	3.413	5.853	0.700	0.861	0.930	0.133	0.258	6.3
	PDT	2.533	5.980	0.775	1.521	0.981	0.053	0.163	9.0
Huang model	CK	4.820	6.739	0.439	0.625	0.991	0.011	0.075	1.6
	NaClO	3.467	5.795	0.723	1.056	0.928	0.111	0.235	6.2
	PDT	2.530	7.149	0.703	0.994	0.980	0.057	0.170	9.0

Notes: CK—control, NaClO—NaClO treatment, PDT—PDT treatment.

**Table 3:** Kinetics model parameters for quality attributes of fresh-cut radishes under different treatments and the predicted quality-based shelf life.

Quality Attributes		Zero-Order			First-Order			Predicted Shelf Life (d)
		*k*	*R* ^2^	*RMSE*	k	*R* ^2^	*RMSE*	
Weight loss	CK	0.415	0.992	0.127	0.309	0.953	0.845	4.8
	NaClO	0.284	0.988	0.108	0.292	0.963	0.557	6.9
	PDT	0.229	0.988	0.295	0.316	0.939	0.348	8.3
Firmness	CK	−21.87	0.962	9.380	−0.041	0.962	11.279	5.5
	NaClO	−19.48	0.999	12.174	−0.035	0.995	14.926	6.7
	PDT	−15.67	0.960	7.034	−0.028	0.947	13.163	7.2
b* value	CK	0.325	0.904	0.624	0.187	0.872	1.254	5.7
	NaClO	0.176	0.956	0.128	0.084	0.968	0.194	4.3
	PDT	0.153	0.968	0.412	0.051	0.943	0.478	6.9
Ascorbic acid	CK	−0.916	0.994	0.580	−0.053	0.999	0.266	5.9
	NaClO	−0.987	0.942	0.836	−0.053	0.98	0.644	6.4
	PDT	−0.829	0.937	0.736	−0.049	0.986	0.320	7.7

Notes: CK—control, NaClO—NaClO treatment, PDT—PDT treatment.

## Data Availability

The original contributions presented in the study are included in the article. Further inquiries can be directed to the corresponding author.
